# Chinese Herbal Medicine Dingji Fumai Decoction for Ventricular Premature Contraction: A Real-World Trial

**DOI:** 10.1155/2020/5358467

**Published:** 2020-04-09

**Authors:** Bo Liang, Fei-Hu Zou, Ling Fu, Hui-Ling Liao

**Affiliations:** ^1^Nanjing University of Chinese Medicine, Nanjing, China; ^2^Tongliang Traditional Chinese Medicine Hospital, Chongqing, China; ^3^Chongqing Traditional Chinese Medicine Hospital, Chongqing, China; ^4^College of Integrated Traditional Chinese and Western Medicine Hospital (T.C.M.) Affiliated to Southwest Medical University, Luzhou, China

## Abstract

**Background:**

Chinese herbal medicine Dingji Fumai Decoction (DFD) is widely clinically used for ventricular premature contraction (VPC). This real-word trial was designed to assess the safety and effectiveness of DFD for VPC.

**Methods:**

This was a double-blinded, randomized placebo-controlled trial. Patients with VPC were randomized (1 : 1) to treatment with DFD combined with metoprolol (DFD arm) or metoprolol combined with placebo (MET arm). A primary end point was a composite of clinical symptoms and signs determined by the traditionalChinese medicine syndrome score and the number of VPC determined by the Holter examination. Second outcomes were adverse events, medication compliance, and laboratory examination.

**Results:**

144 patients were randomized to DFD arm (76 patients) or MET arm (68 patients), and 136 cases (71 in DFD arm and 65 in MET arm) finally completed this trial. After a 12-week follow-up, DFD arm significantly decreased traditional Chinese medicine syndrome score and the number of VPC compared with MET arm (*P* = 0.003 and 0.034, respectively). There was no adverse drug effect and patient medication compliance was good.

**Conclusions:**

Superiority with DFD arm for VPC was demonstrated over MET arm for both the safety and effectiveness end points.

## 1. Introduction

Ventricular premature contraction (VPC) is common, and the incidence increases with age. Although it was 0.6% under the age of 20, the detection rate of 12 lead electrocardiogram was 2.7% over the age of 50 (1). Long-term monitoring showed a 50% incidence of VPC in the population, regardless of the presence or absence of structural heart disease (2, 3). Atherosclerosis Risk in Communities study showed that VPC monitoring in middle-aged patients with or without ischemic heart disease at 2 minutes increased the risk of ischemic heart disease events and death (4, 5). Moreover, the frequency of VPC in the general population is associated with increased cardiovascular risk and mortality (6). In patients without persistent ventricular tachycardia (VT) and structural heart disease, pleomorphic VPC is associated with an increased risk of death and nonfatal cardiovascular adverse events (7). In addition to cardiovascular events, VPC is also associated with stroke (5). As studies have shown, the association between VPC and adverse end point events, VPC, especially pleomorphic and frequent VPC, is considered a risk factor for cardiovascular adverse events and requires treatment to reduce the risk (8). Current pharmacological treatments for VPC include beta-receptor blockers and nondihydropyridine calcium antagonists (9, 10). But CAST showed that antiarrhythmic drugs increased the risk of death in patients with arrhythmia after myocardial infarction (11, 12), the two agents mentioned above can only relieve the symptoms of palpitations in patients (13).

Traditional Chinese medicine (TCM) has been used in clinical practice for more than two thousand years (14), characterizing whole view and syndrome differentiation, and has shown its unique advantages in the prevention, treatment, rehabilitation, and health care of various diseases. TCM has a long history of anti-arrhythmias and great progress has been made in the research on the treatment of arrhythmias in recent years (15–19). Dingji Fumai Decoction (DFD) is an empirical prescription developed by Professor Luo, a national tutor, according to the basic theory of traditional Chinese medicine for the treatment of palpitation (also known as arrhythmia in modern medicine). DFD, consisting of *Chuanxiong Rhizoma* (Chuanxiong), *Jujubae Fructus* (Dazao), *Poria Cocos* (*Schw.*) *Wolf* (Fuling), *Cinnamomi Ramulus* (Guizhi), *Silktree Albizia Bark* (Hehuanpi), *Osdraconis* (*Fossiliaossiamastodi*) (Longgu), *Ostrea Gigas Thunberg* (Muli), *Ziziphi Spinosae Semen* (Suanzaoren), *Radix Polygalae* (Yuanzhi), and *Licorice* (Gancao), is a multi-herbal medicine for ventricular arrhythmia (VA), especially VPC. Its cocomponents with XinSuNing are *Poria Cocos (Schw.) Wolf* and *Licorice*, which significantly restrained arrhythmias induced by the chemical reagents (20). Our previous study showed that DFD has class I antiarrhythmic properties by suppressing Nav_1.5_ dose-dependently with an IC_50_ of 24.0 ± 2.4 mg/mL (21).

So far, it has not been prospectively and systematically tested whether patients with VA can effectively and safely be treated with DFD. The determination of the efficacy and safety of DFD has a potentially high impact on future clinical practice, but science and reasonableness of this strategy has to be assured. The real-world, double-blinded, randomized placebo-controlled trial reported herein the assessed effectiveness and safety of DFD among participants with VPC.

## 2. Methods

### 2.1. Study Design

This was a real-world, double-blinded, randomized placebo-controlled trial conducted at Hospital (T.C.M.) Affiliated to Southwest Medical University, a large tertiary referral center, to assess the safety and effectiveness of DFD combined with metoprolol versus metoprolol alone in symptomatic patients with VPC. The study was approved by the ethics committee at Southwest Medical University (no. 201703-146), and patients provided signed written informed consent before enrollment and were able to withdraw from the study at any time and no explanation was required. This clinical trial was performed according to the revised Declaration of Helsinki 2013 and prospectively registered at Chinese Clinical Trial Registry with a unique identifier ChiCTR-INR-17013548.

### 2.2. Patient Selection

Participants were recruited from January until December 2018. Patients were eligible if they were adults, presented with signs and symptoms of VPC (applied to I ~ IV-A according to the American LOWN classification) in line with the TCM “Heart blood asthenia of palpitation” syndrome with hemodynamic stability and left ventricular ejection fraction ≥50%. To avoid the influence of other Chinese herbal medicines, discontinuation of any form of Chinese herbal medicines for at least 14 days before enrollment was acceptable. Patients were excluded if they were with high mental anxiety or depression, mental illness, and allergic to test ingredients or accessories. The pregnant and lactating women and women planning pregnancy for nearly six months as well as individuals participating in other clinical trials were excluded.

### 2.3. Randomization, Blinding, and Data Quality

After rigorous inclusion and exclusion criteria, patients were randomized to treatment with DFD combined with metoprolol (DFD arm) or metoprolol combined with placebo (MET arm) with a 1 : 1 ratio. Block randomization with computer-generated random number table and sequentially numbered containers each representing a block consisting of ten patients were used for the treatment allocation. Investigators were blinded to treatment assignment and patients remained blinded to treatment assignment throughout the study. Data were monitored for accuracy.

### 2.4. Study Products and Procedure

Chinese herbs that constitute DFD were provided by the Chinese medicine Pharmacy, Hospital (T.C.M.) Affiliated to Southwest Medical University and were identified as authentic by two deputy director pharmacists as described previously (21). All herbs were formed into capsules with a weight of 400 mg by Manufacturing Laboratory, Hospital (T.C.M.) Affiliated to Southwest Medical University. And the placebo capsules were identical in taste, weight, and appearance with the DFD capsules similarly, and all capsules were stored at room temperature. Metoprolol was purchased from AstraZeneca (London, England) as described previously (21). Three different persons generated the random allocation sequence, enrolled the patients, and assigned them to interventions. The participants were instructed into DFD arm consumed DFD 1200 mg *tid* combined with metoprolol 12.5 mg *bid* or MET arm consumed metoprolol 12.5 mg *bid* combined with placebo 1200 mg *tid* for 12 weeks.

### 2.5. Patient Follow-Up

Patients returned for clinical visits at 4, 8, and 12 weeks that included clinical assessment, adverse events, medication compliance, a Holter examination, and laboratory examination.

### 2.6. Outcomes

Primary end point was a composite of clinical symptoms and signs determined by the TCM syndrome score (TSS) and the number of VPC determined by the Holter examination. Second outcomes were adverse events, medication compliance, and laboratory examination. TSS selects 11 different items from the perspective of four TCM diagnoses based on the guidelines for clinical research of new drugs of traditional Chinese medicine issued in 2002 and the guidelines for diagnosis and treatment of common diseases in internal medicine of traditional Chinese medicine issued in 2008 and preliminary investigation and research results (22). Each item has a different classification and corresponding score value, which can be added to obtain the total TSS (Table S1). Patient medication compliance was assessed by counting returned bottles and asking how many doses of the drugs were (or were not) taken.

### 2.7. Statistical Analysis

Continuous data are reported as statistical mean and standard deviation if data subject to a normal distribution, otherwise statistical median and quantile; categorical data are reported as frequency and percentage. Comparisons were performed with independent-samples *t* test, Fisher's exact test, or *χ*^2^ test as appropriate and *P* < 0.050 was considered statistically significant. Time-to-event effectiveness outcomes are displayed through 12 weeks. All analyses were prespecified in a statistical analysis plan. Data were analyzed with SPSS 24.0 (IBM, United States).

## 3. Results

### 3.1. Patient Enrollment and Follow-Up

**Figure 1:**
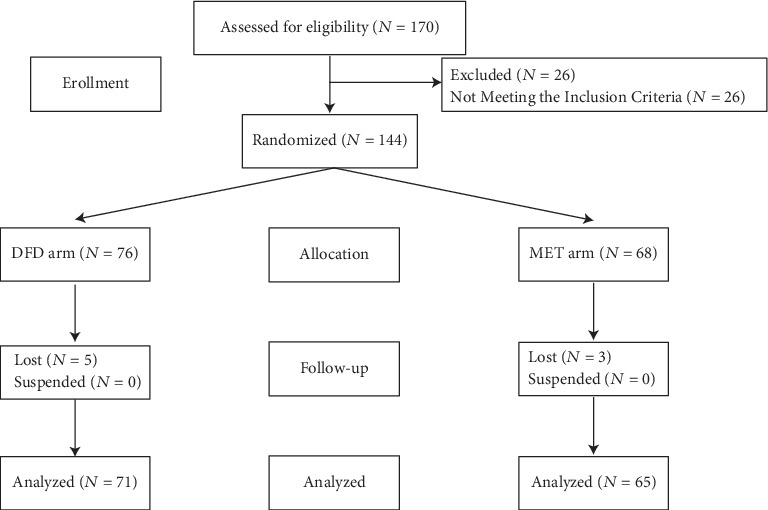
Patient flow diagram.

Between January and December 2018, 144 patients were randomized to DFD arm (76 patients) or MET arm (68 patients) at Hospital (T.C.M.) Affiliated to Southwest Medical University. During 12-week follow-up, 8 cases were lost (5 cases in the DFD arm and 3 cases in the MET arm, respectively) and none was aborted. Finally, 136 cases (71 cases in the DFD arm and 65 cases in the MET arm, respectively) completed this trial (Figure 1).

### 3.2. Patient Characteristics

Randomization proved successful with very similar patient characteristics, cardiovascular diseases, Holter examination, laboratory examination, and TSS profile in the two groups (Table 1).

### 3.3. Primary Outcomes

DFD arm significantly decreased TSS, which can reflect the severity of heart palpitation and the functional condition (*P* = 0.003) and the number of VPC (*P* = 0.034) compared with MET arm. (Table 2).

### 3.4. Second Outcomes

The patients did not report any adverse drug effect. Patient medication compliance was good as no medication was missed or taken by mistake during the trial. Laboratory indicators showed no significant changes from baseline to 12 weeks after treatment.

## 4. Discussion

Drug therapy is often used as the preferred and long-term maintenance treatment in arrhythmias (23). This was the first randomized controlled trial to assess the effectiveness and safety of DFD combined with metoprolol versus metoprolol alone in symptomatic patients with VPC. The trial demonstrated superior safety and effectiveness outcomes in DFD arm and validated the results of the previously reported human study about metoprolol for VPC (24). The effectiveness of metoprolol for VPC was 69.23% in MET arm of this trial, a rate comparable to that observed in a previous study (65%), validating those early promising outcomes. In recent years, research on TCM for VPC has made great progress (16, 25) and has been well received at home and abroad. There are several published randomized trials involving TCM for VPC in similar patient populations and characterized by the same rigorous trial design. A randomized, double-blind, placebo-controlled, parallel-group, multicenter trial (ChiCTR-TRC-12002159) indicated that the Wenxin Keli treatment effectively reduced the overall number of VPC and alleviated VPC-related symptoms in patients without structural heart diseases and had no severe side effects (26). The study used a blank control and a relatively short follow-up (4 weeks), while our present trial chose metoprolol, a recommended medication by guidelines, as a positive control drug and followed up for 12 weeks. A randomized, double-blind, controlled multicenter trial to further evaluate the efficacy and safety of Shensong Yangxin capsule on treating VPC indicated that compared with placebo or mexiletine, Shensong Yangxin capsule has significant therapeutic efficacy in reducing VPC numbers and alleviate VPC-related symptoms (27). A systematic review of randomized controlled trials involving 2441 patients showed that Zhigancao Decoction appears to have beneficial effects on the improvement of total effects and reduction of the number of VPC (28). However, in many cases, one arrhythmia does not appear alone and is often associated with other types of arrhythmias or other heart diseases and cardiovascular diseases. A systematic review and meta-analysis of randomized clinical trials showed that Wenxin Keli may be effective and safe for treating VPC with heart failure (29). A randomized, double-blind, multicenter clinical trial (ChiCTR-TRC-12002061, NCT01612260) showed that in this 12-week pilot study, Shensong Yangxin capsule was demonstrated to have the benefits of VPC suppression and cardiac function improvement with good compliance on a background of standard treatment for congestive heart failure (30). A double-blind, placebo-controlled, multicentre, randomized clinical trial (NCT01750775) involving 333 patients showed that Shensong Yangxin capsule is an effective antiarrhythmic therapy for symptomatic, frequent VPC uniquely suited patients with concomitant sinus bradycardia (31). In our real-word study, in addition to VPC, VT and atrial arrhythmia were also detected in patients and about half of the patients with coronary heart disease (CHD), DFD can still show significant efficacy. Since CHD can also cause arrhythmia (32), we have not observed relevant indicators of CHD in patients to explore whether VPC treated by DFD is independent from CHD or caused by CHD, which needs to be verified by rigorous clinical trials in the later stage. But overall, DFD works, which is encouraging.

TSS is widely used in TCM clinical research (33, 34). We innovatively applied TSS to this study, which was not found in other related studies (26, 27, 30, 31). Patient medication compliance has a certain effect on the therapeutic effect of the disease. This trial assessed test patient medication compliance by counting returned bottles and asking how many doses of the drugs were (or were not) taken and no medication was missed or taken by mistake during the trial, indicting patient medication compliance was good in our study. Serious mental illness can affect various diseases, including cardiovascular health (35). Studies demonstrated addressing the mental health of patients may improve patient-reported outcomes (36). Mental anxiety or depression is associated with cardiovascular processes and cardiorespiratory fitness (37–39), diastolic dysfunction, additionally, is a common finding among depression patients (40). Thus, we excluded hereby patients with high mental anxiety or depression (self-rating anxiety scale score above 70 or self-rating depression scale score above 73). Several limitations of this study deserve further discussion. Although metoprolol was used as a positive control drug, the dose was not adjusted for specific patient conditions. Another point is that this real-world, double-blinded, randomized placebo-controlled trial is ultimately a single-center trial, involving only the Chinese population. Given the increasing popularity of TCM throughout the world, rigorously designed, multi-center, long-term, large-scale, and methodologically sound trials are warranted.

## Figures and Tables

**Table 1:** Demographic and baseline clinical characteristics.

	All (*N* = 136)	MET arm (*N* = 65)	DFD arm (*N* = 71)	*P*
Male	65 (47.8%)	33 (50.8%)	32 (45.1%)	0.51

Age	63 ± 11	65 ± 12	62 ± 11	0.15

Cardiovascular diseases				
CHD	72 (52.9%)	39 (60.0%)	33 (46.5%)	0.10
HHD	33 (24.3)	16 (24.6%)	17 (23.9%)	
VHD	12 (8.8%)	5 (7.7%)	7 (9.9%)	
PHD	12 (8.8%)	3 (4.6%)	9 (12.7%)	
DCM	2 (1.5%)	2 (3.1%)	0 (0.0%)	
Others	2 (1.5%)	0 (0.0%)	2 (2.8%)	
None	3 (2.2%)	0 (0.0%)	3 (4.2%)	

Holter examination				
Single VPC	1276 (877-4257)	1241 (851-2861)	1426 (885-5454)	0.23
Double VPC	0 (0-4)	1 (0-4)	0 (0-9)	0.98
Triple VPC	0 (0-3)	0 (0-3)	0 (0-3)	0.76
Paired VPC	3 (0-24)	2 (0-17)	3 (0-45)	0.33
VT	0 (0-0)	0 (0-0)	0 (0-1)	0.02
Single APC	218 (0-1651)	184 (0-1044)	247 (0-2569)	0.32
AT	1 (0-17)	1 (0-13)	1 (0-21)	0.26
AF	0 (0-0)	0 (0-0)	0 (0-0)	0.49

Laboratory examination				
WBC (∗10^9^/L)	7.6 (5.3-9.1)	7.7 (5.4-9.2)	7.4 (5.3-9.1)	0.35
RBC (∗10^12^/L)	4.26 (3.35-5.21)	4.27 (3.42-5.12)	4.20 (3.05-5.27)	0.47
PLT (∗10^9^/L)	105 (95-111)	102 (94-109)	107 (97-114)	0.02
ALT (U/L)	33 (17-49)	38 (17-52)	32 (18-46)	0.28
AST (U/L)	35 (23-49)	34 (24-51)	35 (21-48)	0.91
TBIL (*μ*mol/L)	17.4 (11.0-24.3)	15.7 (10.1-24.3)	19.3 (11.5-24.3)	0.33
TP (g/L)	74.0 (64.6-82.8)	72.8 (63.6-82.1)	74.0 (66.6-83.4)	0.59
BUN (mmol/L)	6.40 (4.04-8.76)	6.58 (4.05-7.94)	6.35 (4.03-9.25)	0.60
Cr (*μ*mol/L)	79 (63-96)	80 (67-96)	78 (62-96)	0.31
UA (*μ*mol/L)	327 (270-401)	325 (270-395)	328 (268-412)	0.74

TSS	18 ± 3	18 ± 3	17 ± 3	0.48

Abbreviation: MET arm: metoprolol combined with placebo; DFD arm: Dingji Fumai Decoction combined with metoprolol; CHD: coronary heart disease; HHD: hypertensive heart disease; VHD: valvular heart disease; PHD: pulmonary heart disease; DCM: dilated cardiomyopathy; VPC: ventricular premature contraction; VT: ventricular tachycardia; APC: atrial premature contraction; AT: atrial tachycardia; AF: atrial fibrillation; WBC: white blood cell; RBC: red blood cell; PLT: platelet; ALT: alanine aminotransferase; AST: aspartate aminotransferase; TBIL: total bilirubin; TP: total protein; BUN: blood urea nitrogen; Cr: creatinine; UA: uric acid; TSS: traditional Chinese medicine syndrome score.

**Table 2:** Effectiveness outcomes.

		All	MET arm	DFD arm	*χ* ^2^	*P*
VPC	Valid	105	45	60	4.500	0.034
	Invalid	31	20	11		

TSS	Valid	117	50	67	8.591	0.003
	Invalid	19	15	4		

Abbreviation: MET arm: metoprolol combined with placebo; DFD arm: Dingji Fumai Decoction combined with metoprolol; VPC: ventricular premature contraction; TSS: traditional Chinese medicine syndrome score.

## Data Availability

A portion of the relevant data used to support the findings of this study are included within the supplementary information file and another portion were supplied by Hui-Ling Liao under license and so cannot be made freely available. Requests for access to these data should be made to Hui-Ling Liao (liaohl@swmu.edu.cn).

## Abbreviations

CHD: Coronary heart disease
DFD: Dingji Fumai Decoction
TCM: Traditional Chinese medicine
TSS: Traditional Chinese medicine syndrome score
VA: Ventricular arrhythmia
VPC: Ventricular premature contraction
VT: Ventricular tachycardia.

## Supplementary Materials

Supplementary 1 Table S1. Traditional Chinese medicine syndrome score with Heart blood asthenia of palpitation.
