# Supplementary material for: Chinese Herbal Medicine Dingji Fumai Decoction for Ventricular Premature Contraction: A Real-World Trial
Source: Biomed Res Int. 2020 Apr 9;2020:5358467. doi: 10.1155/2020/5358467 (PMC7171618; doi:10.1155/2020/5358467)
Supplement: Supplementary 1 — Table S1. Traditional Chinese medicine syndrome score with Heart blood asthenia of palpitation. [file 5358467.f1.pdf]

Table S1. TSS with heart blood asthenia of palpitation

Items		Classification				Score
Primary Symptoms	Palpitation	0	2	4	6	
	Dizzy	0	2	4	6	
	Complexion	0	2	4	6	
	Shortness of Breath	0	2	4	6	
Secondary Symptoms	Languid	1	2	3	4	
	Poor Appetite	1	2	3	4	
	Forgetfulness	1	2	3	4	
	Insomnia	1	2	3	4	
Tongue Manifestation	Tongue Reddish	0		2		
	Less Fur	0		2		
Pulse Manifestation	Gracility	0		2		
Total						

Note: The primary symptoms were divided into none, mild, moderate and severe four classifications, and then scored 0, 2, 4 and 6, respectively. Again, the secondary symptoms were also classified into 4 grades and scored 1, 2, 3 and 4, respectively. For tongue and pulse manifestation, there were only two grades: with and without, and marked 2 and 0, respectively.
